# Basilar invagination associated with chiari malformation type I: A literature review

**DOI:** 10.6061/clinics/2019/e653

**Published:** 2019-04-04

**Authors:** José Nazareno Pearce de Oliveira Brito, Bruna Afonso dos Santos, Isys Fialho Nascimento, Leonardo Augusto Martins, Cléciton Braga Tavares

**Affiliations:** I Departamento de Neurologia e Neurocirurgia, Faculdade de Ciencias Medicas (FACIME), Universidade Estadual do Piaui, Teresina, PI, BR.; II Centro Universitário UNINOVAFAPI, Teresina. PI, BR.; III Hospital Sao Marcos, Teresina, PI, BR.

**Keywords:** Craniovertebral Junction, Basilar Invagination, Chiari Malformation, Surgical Decompression

## Abstract

Basilar invagination (BI) and Chiari malformation type I (CM-I) are very important anomalies that introduce instability and compression in the occipitocervical transition region and have complex clinical characteristics. These anomalies vary according to the affected structures. The present study revises current knowledge regarding the anatomy, anatomo-physiology, clinical manifestations, and radiological findings of these entities and the associated surgical treatment approaches. A bibliographic survey was performed through a search in the Medline, PubMed, SciELO, Science and LILACS databases. When associated, these craniovertebral malformations result in neurological deficits due to neural parenchyma compression; however, the presence of microtraumas due to repetitive lesions caused by the bulb and cervical marrow instability has been highlighted as a determinant dysfunction. Surgical treatment is controversial and has many technical variations. Surgery is also challenging due to the complex anatomical characteristics and biomechanics of this region. Nevertheless, advances have been achieved in our understanding of related mechanisms, and compression and atlantoaxial instability are considered key elements when selecting the surgical approach.

## INTRODUCTION

Basilar invagination (BI) is an important occipitocervical malformation characterized by odontoid apophysis displacement of the axis inwards towards the foramen magnum and posterior displacement of the bulb; cerebellar BI is generally a congenital pathology, but it can be acquired in rare cases (1–3). This malformation was first described by Ackermann (4) in studies on cretinism performed in the Andes, but this description was subsequently rejected by Virchow (5), who referred to the malformation as basilar impression malformation, thoroughly described the bone changes observed in odontoid process invagination at the base of the skull, and was the first author to accept a congenital origin for the pathological and clinical characteristics. Those results were reaffirmed by Homén (6).

Chiari (7) described changes in the inferior position of the cerebellar tonsils and the medial regions of the lower cerebellar lobes, which moved through the craniospinal transition towards the upper spinal canal >5 mm; this condition is currently known as Chiari malformation type I (CM-I).

The incidence of CM-I is estimated to be 1/1,000 births (8). In rare cases, CM-I is complicated by associations with other malformations of the craniocervical junction, including mainly BI (1,8,9).

Craniometric studies have shown that such malformations present common characteristics, including underdevelopment of the occipital bone and compression of cerebrospinal fluid (CSF) at the craniocervical junction (1,8,10).

Even when congenital, these interrelated changes can trigger symptoms in adulthood, and the complex clinic al characteristics vary in accordance with the involved structures. Clinical manifestations, either when isolated or combined, can include headache, neck pain, neck stiffness, diplopia, dysphagia, rhinolalia, paresthesia of the face and limbs, vertigo, sexual disorders and progressive cerebellar ataxia (1,11).

Treatment is complex, and the surgical approach varies according to the condition of the craniovertebral malformations (2,9,12). However, the therapeutic focus has facilitated advances in our understanding of these occipitocervical anomalies (12).

The aim of this study was to review the anatomo-physiology, clinical manifestations, and radiological findings of BI associated with CM-I and the corresponding surgical approaches.

## METHODS

This is a review of BI and CM-I. A bibliographic survey was carried out in the Medline, PubMed, SciELO, Science and LILACS databases. Articles ranged from early records of the malformations in question to the most current approaches to this topic. The descriptors used were *“Craniovertebral Junction, Basilar Impression, Chiari Malformation, Surgical Decompression”* and their correspondents in other languages, either alone or in combination.

The inclusion criteria were as follows: original articles regarding the association between BI and CM-I corresponding to publications on anatomical aspects of the craniocervical region, vascular relationships, types of compression, radiological findings and types of surgical approaches. The exclusion criteria included articles that reported none of the previously mentioned correlations, those focusing on other types of Chiari malformation, and/or duplications among the databases.

All the bibliographic material included in this review was analyzed by critically reading the data to frame the inclusion criteria and interpret and compare the findings.

## RESULTS

### Anatomical changes in the craniocervical region and their vascular relationships

Anatomic recognition of the craniocervical region (an area with many pathological inclinations) becomes indispensable in acute basilar impressions with CM-I.

To simplify our study, the craniocervical junction included only the clivus, foramen magnum, occipital condyles and upper cervical vertebrae (atlas and axis), which, apart from their function as joints, surround and protect the arterial and venous vascular tree as well as nervous structures (bone marrow, bulb, spinal nerves and craniocaudal structures) within the craniovertebral subarachnoid space. Knowledge of the muscles surrounding this site and the vessels adjacent to the spinal cord, in addition to the bulb and the nerves that operate in the region, is essential when performing various surgical approaches (13–20).

In particular, with BI, anatomical changes can occur in the foramen magnum, clivus and upper cervical spine. Skeletal defects in this region affecting the odontoid process (axis) and the clivus have been observed, reflecting various types of BI (13–15).

– Anterior basilar impressions have been found in clivus hypoplasia with shortening of the posterior base of the skull but without alterations in the occipital foramen (13).
– Medial basilar impressions can move upwards from the anterior wall of the foramen magnum, and the condyles can slide medially against the lateral parts of the condyles at the base of the skull (13). When this anomalous movement of the bone defect occurs, part of the occipital bone and the odontoid process invaginates into the craniovertebral region, resulting in considerable narrowing of the anatomical and subarachnoid spaces and thus causing impairment of vital nerve structures, such as the bulb, upper spinal cord, cranio-caudal nerves and upper cervical roots, which can substantially and negatively impact the vertebral artery circulation, the posterior inferior cerebellar artery and its inferior, lateral and posterior medullary branches. Damage to the meningeal branches and anterior spinal artery can also impair the flow of the venous drainage system through the anterior, lateral and posterior veins of the bulb, vermis inferior vein and root veins, which can result in circulatory disorder of the spinal fluid flow (14,17,20).

### Types of compression and anatomo-physiological and clinical alterations

The association of BI with CM-I invagination syndrome may result in neurological deficits due to neural parenchyma compression because of tonsillar herniation or due to hydrodynamic changes in the CSF at the level of the craniovertebral junction. However, an isolated measurement of the invagination depth will not necessarily be correlated with the severity of neurological symptoms, and patients with symptoms similar to CM-I have been found to have less than five millimeters of hernia (10).

In 2009, Goel and Shah (21) suggested that in BI, physical and musculoskeletal alterations are secondary to the instability and compression of compromised neural structures and should be corrected after decompression and stabilization. Goel (22) also called attention to a determining factor that should be considered in the pathophysiology of IB: the presence of microtraumas due to repetitive lesions originating from instability. In the same study (22), he observed that more severe instability was associated with a more acute clinical presentation and worse neurological deficits.

The vascular network modifications in the posterior fossa observed in cases of BI and CM-I likely account for most pathophysiological neurological and clinical signals as well as postoperative complications because they can lead to disturbances in the structures that supply blood to this region. In these cases, the posteroinferior cerebellar artery is likely to have an abnormal position and descend in the caudal direction, and it may or may not accompany cerebellar tonsil herniation (23).

In contrast, in Chiari syndrome I, elongation of the cerebellar tonsils around the bulb has been observed, with these structures descending together with the spinal canal. This pressure within the spinal canal in the presence of a basilar impression further reinforces the functional loss of CSF circulation, generating real signs of hydrocephalus, syringobulbia and syringomyelia as consequences of increased intracranial cerebrospinal pressure (13). In adults, sensitive cervical and cranial nerve disorders are frequently observed. Over time and with symptom persistences, impairments of the long sensory and motor pathways can occur, causing paraparesis, diparesis, and sexual impotence due to impairment of the spinal cord posterior pathways (13–20).

Among other etiologies (24), syringomyelia formations related to CM-I are believed to occur with the reduction in CSF in the IV ventricle. Mechanical blocks caused by the reduced capacity of the posterior fossa associated with inferior displacement of the cerebellar tonsils can alter the velocity and volume of CSF flow in the craniocervical transition and result in consequential syrinx medullary enlargement (25–28).

### Radiological findings, signals and symptoms

The first reports of BI were based on postmortem findings (12). In 1911, Schuller (29) made the first radiological diagnosis in live patients, and those findings were further corroborated by Chamberlain and other radiologists.

**Figure 1:**
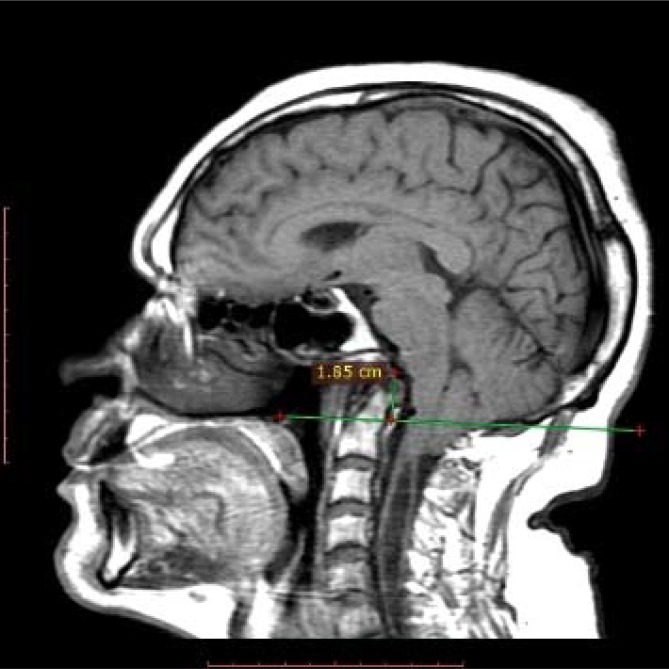
The odontoid process crosses the Chamberlain line for 18.5 mm and compresses the pons anteriorly.

**Figure 2:**
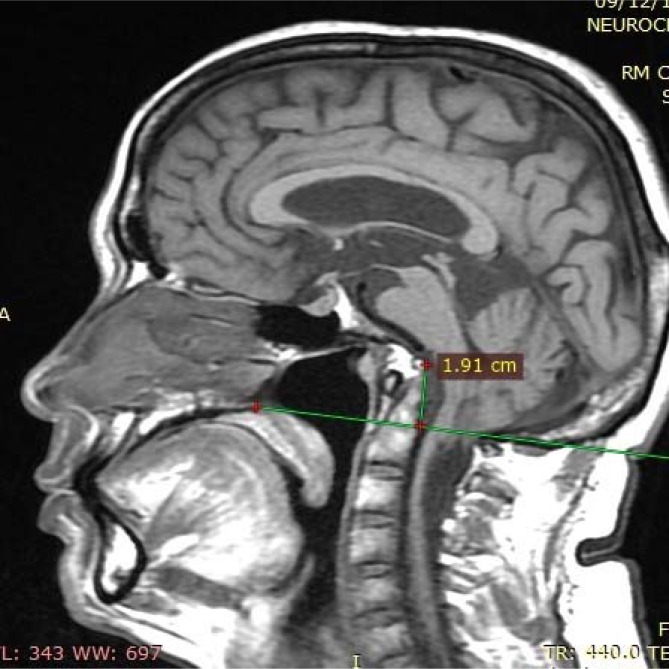
The odontoid process crosses the Chamberlain line for 19.1 mm (basilar invagination) and is associated with the clivus, compressing the pons and bulb anteriorly.

In 1939, Chamberlain (30) defined a method for preoperative diagnosis based on the breach of the line of the skull base; however, his criterion was controversial among scholars in the field, and its detection on radiography was cumbersome. In current practice, however, BI is more commonly diagnosed because of advances in neuroimaging examinations (2,31) (Figures 1 and 2). Most patients (60-80%) have posterior headache or neck pain. However, other more consistent symptoms may occur due to brainstem compression or cranial nerve dysfunction (32,33). The association with CM-I is common and has been reported to have a frequency of more than 90% and a high incidence and prevalence in northeast Brazil in the literature (31,34,35,36).

Imaging methods, such as X-ray, computed tomography, magnetic resonance imaging and other examinations used to evaluate these malformations, have shown that the volume of the posterior fossa is lower in patients with BI and CM-I than that in normal individuals. Milhorat et al. (37) detected a decrease of 13.4 ml in the volume of the posterior fossa and a 40% (10.8 ml) reduction in CSF volume in the posterior fossa (38).

**Figure 3:**
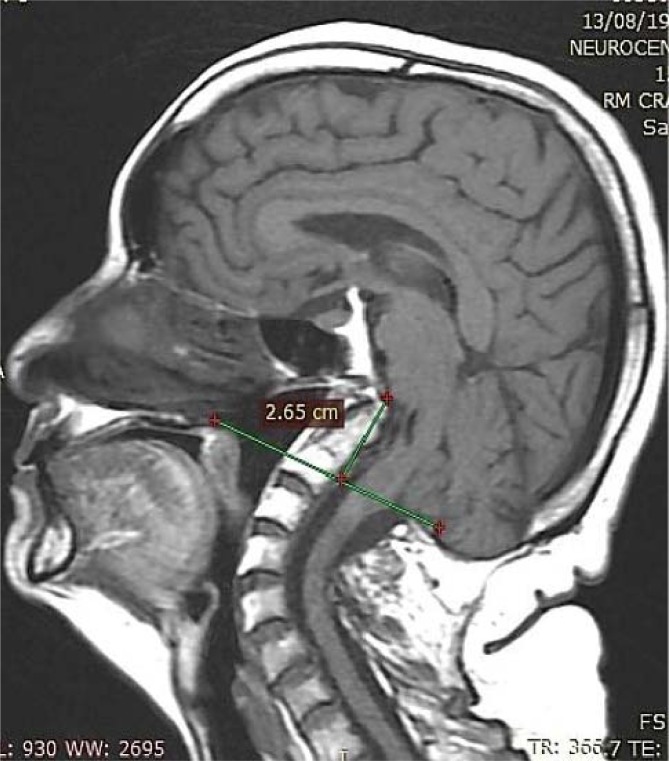
The clivus is shortened, and the upper extremity of the odontoid process lies 26.5 mm across the Chamberlain line, consistent with basilar invagination, and compresses the pons anteriorly.

**Figure 4:**
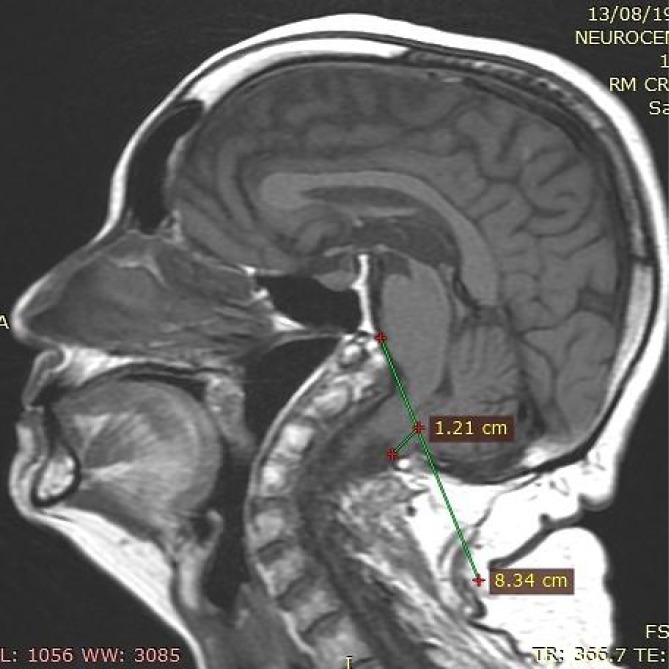
The cerebellar tonsils migrated 12.1 mm towards the foramen magnum and compressed the bulb posteriorly, consistent with Chiari type I malformation.

The measured caudal dislocation of the cerebellar tonsils represents crucial information for a diagnosis. Ectopia should be measured in median sagittal resonance imaging (Sequence T1, T2 or three-dimensional (3D) Constructive Interference in Steady State (CISS), or another) from a line drawn on the foramen magnum running from the *basion* to the *opisthion*. However, the normal limit of tonsillar migration remains controversial, with the normal limit considered a distance smaller than 3 mm, a borderline limit between 3 and 5 mm, and a pathological limit higher than 5 mm. Some authors consider herniation of 3 mm pathological if accompanied by typical clinical symptoms, such as lengthening of the cerebellar tonsils, syringomyelia, or an abnormally elongated IV ventricle (32,33) (Figures 3 and 4).

Less frequently, cerebellar tonsil herniation can be accompanied by medullar-bulb kinking, similar to that typically observed in Chiari malformation type II. These cases are described as bulbar or a myelencephalic variant of CM-I, which is also called type 1,5 (39–41). This type of malformation is likely associated with a more substantial reduction in posterior fossa dimensions, and other associated skeletal alterations often exist, such as platibasia, BI or hypoplasia of the occipital condyles.

Hydrosyringomyelia is frequently associated with the symptoms of these patients and their prognoses; therefore, the entire spinal cord must be evaluated using imaging exams, specifically, magnetic resonance imaging. On the other hand, patients with an initial hydrosyringomyelia diagnosis should be assessed by imaging exams to visualize the cervicocranial transition and eliminate the possibility of CM-I (40,41).

The sequences of magnetic resonance images obtained using phase contrast flow sensitivity have demonstrated noninvasively that dynamic changes occur in the liquoric flow through the foramen magnum in patients with CM-I, which can assist in the evaluation of the benefits of decompressive surgery. The findings obtained in these patients are still controversial; some authors have demonstrated selective liquoric flow skull obstruction to the vertebral canal during systole and normal craniovertebral diastolic flow, while others have shown increased spinal cord mobility in the phases of systole (downward) and diastole (upward), with impaired liquoric flow in the diastolic phase. Currently, however this liquoric flow study is not routinely used in therapeutic planning in these patients (42).

### Types of surgical approaches and their indications

To institute appropriate BI surgical treatment in cases associated with or without malformations, a better understanding of the pathogenesis of this condition is needed. Ebenius (43) reported a surgical treatment for a basilar impression first performed by Olivecrona in 1932.

In 1939, Chamberlain (30) reported on four patients with BI for whom surgical treatment via decompressive craniectomy with cervical laminectomy and dural opening was suggested for management to relieve compression in the craniocervical junction. However, in the same period, Vet (44) questioned the usefulness of this suboccipital decompression technique in asymptomatic cases.

In the following years, patients were treated for craniovertebral abnormalities with decompression of the posterior fossa by enlargement of the opening of the foramen magnum and C1 arch removal. Since then, the optimal treatment for symptomatic cases of BI and/or CM-I is considered surgery with opening of the dura mater (3, 45).

Attempts to use conservative treatment have not produced convincing results according to a report by Phillips (46) in 1955.

In northeast Brazil, Caetano de Barros et al. (3) documented a study of 66 cases submitted to foramen magnum decompression and arch decompression of the atlas and axis, and the results suggested that performing dural opening produced satisfactory results in patients with progressive symptoms.

Tenuto et al. (47) published a study describing posterior fossa decompression with opening and postsurgical preservation of the pachymeninges. However, the morbidity and mortality in the patients were high due to the possibility that scars had formed in the leptomeninges or tonsillar herniation had occurred (45,48).

In 1977, Gonçalves da Silva (49) applied technical modifications to BI surgical treatment for patients with indications for dura mater plastic surgery to increase the anatomical space of the posterior fossa, especially in cases associated with CM-I, to prevent a liquoric fistula and rebuild pachymeningial integrity, thus offering greater protection to nervous structures in this region (45,50).

In a study on atlantoaxial instability in rheumatic inflammatory processes entitled “Basilar Pseudo invagination”, Brito et al. (15) reported that decompression with posterior fixation is unavoidable, and they recommended this procedure in BI for joint instability.

In BI cases in which the atlantoaxial joint with odontoid process protrusion and brainstem compression are considered fixed or irreducible, anterior decompressive surgery becomes a treatment priority, which has shed light on the events occurring during the years following surgery involving odontoid apophysis resection using a transoral approach (51,52). This procedure was performed successfully in vivo for the first time by Roberts in 1965 and was mentioned by Grote et al. (53), who also carried out this operation in 1971 and in 1972 and proposed that this should be the treatment of choice for these cases (53).

Menezes et al. (54) proposed guidelines for the surgical approach to craniocervical abnormalities after considering the types of compression and the possibility of reduction. For reducible craniovertebral abnormalities, posterior fixation was recommended; however, for irreducible or fixed abnormalities, the treatment algorithm was based on the location of compression in the ventral and dorsal groups. For stable ventral pathologies, only transoral decompression was indicated, while for unstable pathologies, this procedure was followed by posterior occipitocervical fixation. Similarly, for dorsal pathologies, decompression was recommended with or without stabilization.

Goel and Laheri (55) expanded the methods used for surgical approaches to craniovertebral pathologies with lateral mass fixation of the atlas and axis (56).

In 1998, Goel et al. (57) evaluated the best surgical treatments in 190 patients diagnosed with BI according to Chamberlein's criteria and divided the patients into two groups. Group I consisted of 88 patients who presented odontoid process invagination in the foramen magnum and brainstem compression. Group II exhibited basal impressions with CM-I and reduced cranial fossa volume (57). They used transoral resection of the odontoid process in Group I patients and foramen magnum decompression in the patients in Group II because these were selected as the best treatment choices, and atlantoaxial or occipitocervical fixation was subsequently performed as necessary in more patients in Group I than in Group II (57).

In 2005, Kassam et al. (58) performed endoscopic transnasal odontoidectomy and demonstrated the feasibility of this approach. With the development of endoscopic technology, this approach has been successfully performed to treat BI with cervicomedullary compression (59).

In 2009, Goel and Shah (21) reported a study of 170 patients with possible reversal of long-standing musculoskeletal changes after decompression and stabilization of the craniovertebral junction.

In 2011, da Silva et al. (31) showed promising clinical results related to the management of 104 patients BI or CM with or without syringomyelia who were subjected to surgical treatment in the seated position with craniectomy, tonsillectomy, fourth ventricle opening and duraplasty to increase the size of the cisterna magna.

To improve our understanding of BI mechanisms and the ability to carry out treatment, Goel (51) proposed the concept that atlantoaxial displacement or instability should be the primary pathogenetic factor in all types of BI associated with or without CM-I. Goel then proposed a radical treatment strategy with craniovertebral realignment and arthrodesis instead of decompression and bone removal (51,60,61). As a contribution, those findings reaffirmed the instability theory (62), and in these cases, foramen magnum decompression would not be necessary and may even have no long-term effect.

## CONCLUSION

Considering the embryonic development of the bone and central nervous system, a malformation can occur in either one of these components or in both systems. For example, differences exist in interpretations of BI pathogenesis associated with Arnold-Chiari malformation, and this serves to illustrate the difficulty in establishing the priority of the nervous or bone system. In fact, several theories about how these damages originate exist.

For a long time, BI was viewed as an anatomical and radiological phenomenon requiring clarification. However, in recent years, this disease has entered the field of neurosurgery. With the advent of computerized imaging, particularly helicoidal CT with bone reconstruction and magnetic resonance imaging, demonstrating the anatomical and biomechanical peculiarities and describing the compromised vascular relationships of the craniovertebral region in these malformations have become possible.

Advances in research in the area have also provided us with a better understanding of the pathophysiological mechanisms and profiles of the clinical presentations of these patients, which is essential to directing surgical treatment. Considering BI/CM-I without instability or posterior compressions, symptomatology resolution benefits from simple posterior decompression, in cases with instability cranial-cervical or atlantoaxial, fixation is necessary. Using the anterior approach (transoral or nasal endoscopy) in patients with brain stem compression without instability, and posterior arthrodesis is then reserved for patients with functional instability.
